# Impact of post stroke depression and anxiety on health-related quality of life in young Filipino adults

**DOI:** 10.3389/fstro.2023.1149406

**Published:** 2023-03-16

**Authors:** Katrina Hannah D. Ignacio, Jose Miguel M. Medrano, Sitti Khadija U. Salabi, Alvin J. Logronio, Sedric John V. Factor, Sharon D. Ignacio, Jose Leonard R. Pascual, Maria Carissa Pineda-Franks, Jose Danilo B. Diestro

**Affiliations:** ^1^Department of Neurosciences, Philippine General Hospital, College of Medicine, University of the Philippines Manila, Manila, Philippines; ^2^Department of Clinical Neurosciences, University of Calgary, Calgary, AB, Canada; ^3^Critical Care Services, St. Luke's Medical Center, Quezon City, Philippines; ^4^Department of Family and Community Medicine, Philippine General Hospital, College of Medicine, University of the Philippines Manila, Manila, Philippines; ^5^Department of Medical Imaging and Therapeutic Radiology, National Kidney and Transplant Institute, Quezon City, Philippines; ^6^Department of Psychiatry and Behavioral Medicine, Philippine General Hospital, College of Medicine, University of the Philippines Manila, Manila, Philippines; ^7^Department of Rehabilitation Medicine, Philippine General Hospital, College of Medicine, University of the Philippines Manila, Manila, Philippines; ^8^Department of Medical Imaging, Division of Diagnostic and Therapeutic Neuroradiology, St Michael's Hospital, University of Toronto, Toronto, ON, Canada

**Keywords:** post stroke anxiety, post stroke depression, health-related quality of life, HRQOL, EQ-5D-5L, young adults, Filipinos

## Abstract

**Background:**

Health-related quality of life (HRQoL) is important to assess in young adults who suffer from various physical and mental consequences after stroke. We aimed to evaluate the HRQoL of young adults after ischemic or hemorrhagic stroke and to determine the association of anxiety and depression with poor HRQoL in this special population.

**Methods:**

We administered the European Quality of Life Five Dimension Five Level Scale (EQ-5D-5L) to assess the HRQoL in our study population. This tool describes health outcomes in five dimensions. Socio-demographic and clinical data including modified Rankin scale (mRS), Barthel Index and Hospital Anxiety and Depression Scale scores were available from our previous cross-sectional study on young adults with stroke. We performed bivariate analyses to assess the association of psychiatric comorbidities with categorical characteristics and determined risk factors for poor HRQoL using multivariable logistic regression analysis.

**Results:**

We evaluated HRQoL, psychiatric and functional outcomes in 114 young adult stroke patients. CVD infarct was more common than hemorrhage (58.8 vs. 41.2%). Patients with both anxiety and depression were found to have the lowest ratings on the HRQoL scales, with an EQ VAS of 60 vs. 90 (*p* = 0.01) and an EQ Index of 0.64 vs. 0.89 (*p* < 0.01) when compared to those without both conditions. Anxiety and depression were significantly correlated with poor quality of life on all dimensions of the EQ-5D-5L. Similarly, Barthel Index was a significant predictor for problems in HRQoL (OR 0.17, 95% CI 0.03–1.02 on the mobility dimension and OR 0.08, 95% CI 0.01–0.55 on the self-care dimension). Cerebral hemorrhage was an independent predictor for poorer self-care dimension scores (OR 4.99, 95% CI 1.42–17.56).

**Conclusions:**

Our study showed that anxiety, depression and poor functional status are associated with poorer HRQoL in young adult Filipinos after stroke. Screening for psychiatric conditions and evaluating mobility are crucial in the management of this special population after stroke.

## 1. Introduction

Stroke is the second most common cause of disability adjusted life years (DALYs) affecting more than 80 million stroke survivors worldwide (Johnson et al., 2019). The impact of stroke is disproportionately higher in young adults who are left disabled during their most productive years (Smajlovic, 2015; Ekker et al., 2019). Health-related quality of life (HRQoL) which evaluates the physical, mental and social health of patients is an important outcome to measure. Though an increasing number of epidemiological studies on stroke in young adults have been published in recent years, there are fewer studies that have focused on HRQoL in this special population (Smajlovic, 2015; Bartholomé and Winter, 2020).

In low-middle income countries, the incidence of stroke is expected to rise even further as they face challenges in addressing modifiable vascular risk factors (Johnson et al., 2019). In 2014, a local study on the burden of stroke in the Philippines estimated that half a million Filipinos will be affected by stroke (Navarro et al., 2014). The age-standardized DALY rate for stroke also rose by 15.7% from 1990 to 2016 (Johnson et al., 2019). This poses a special challenge to stroke patients in the country since funding for healthcare is largely out of pocket (Navarro et al., 2014).

The Philippines faces gaps in relevant knowledge and epidemiologic data on the outcome of stroke patients. Only one local study that evaluated the HRQoL in stroke survivors was found (Valeros, 2017). The study showed mobility to be the most affected domain in stroke survivors and emphasized the need for local studies in determining HRQoL of stroke patients (Valeros, 2017). In a more recent study, we found that young Filipino adults are at considerable risk for depression and anxiety after stroke (Ignacio et al., 2022). Similar studies from other countries have shown that these psychiatric conditions are associated with a poorer quality of life among stroke survivors (Su et al., 2014; Chun et al., 2018).

Provision of stroke care services requires epidemiologic studies and continued data collection on outcomes of stroke patients (De Ryck et al., 2013). Our study hopes to address the gaps in health service delivery and knowledge on stroke epidemiology and outcomes in the Philippines. Data on HRQoL and risk factors for poor HRQoL in stroke patients are critical in improving stroke care services.

Our study aimed to describe the health-related quality of life in young adult Filipinos who suffered from stroke using the EQ-5D-5L scale. We further aimed to determine the association of anxiety and depression with the various dimensions of the EQ-5D-5L scale.

## 2. Methods

### 2.1. Study design

We included young adult stroke survivors from 2019 to 2021 who participated in a single-center cross-sectional epidemiological study that had been previously published (Ignacio et al., 2022). The study protocol was approved and endorsed by the University of the Philippines Manila—Research Ethics Board through the Office of the Philippine General Hospital-Expanded Hospital Research Office (Code: 2019 270-01). Written informed consent was obtained from each participant. The study followed the Strengthening the Reporting of Observational Studies in Epidemiology (von Elm et al., 2007).

### 2.2. Patient selection and data collection

We included patients consulting at the outpatient clinics of Adult Neurology and Rehabilitation Medicine (1) aged 18–49 at the time of stroke; (2) between 21 days up to <2 years from stroke; (3) diagnosed with ischemic stroke or intracranial hemorrhage as established by cranial CT or MRI. Patients with severe cognitive deficits, severe aphasia and personal history of depression or anxiety were excluded. The final patient population included all 114 patients in our originally described cohort. The following clinical information were available for the cohort: modified Rankin scale (mRS) to quantify disability due to stroke, Barthel Index (BI) to measure independence in activities of daily living and the Mini Mental State Exam (MMSE) to measure cognitive functioning. The Hospital Anxiety and Depression Score—Pilipino version (HADS-P) scores for the population were also available. A cut-off of ≥7 points was applied for the depression subscale and ≥7 points for the anxiety subscale (De Guzman, 2013).

### 2.3. Structured survey tool

We administered a structured survey tool to eligible patients. The tool was designed to characterize the study population in accordance to the study's objectives. Items included age, sex, civil status, address, educational attainment, occupational status, and income.

### 2.4. Determination of health-related quality of life

We administered the Filipino (Tagalog) version of the European Quality of Life Five Dimension Five Level Scale (EQ-5D-5L) to evaluate the health-related quality of life in our sample population during the interviews for anxiety and depression (conducted from 2019 to 2021). The EQ-5D-5L is a self-administered tool that describes health outcomes in five dimensions: mobility, self-care, usual activities, pain or discomfort and anxiety or depression. The study was registered under the EuroQol Research Foundation where the translated version of the tool was requested for use in the study (EuroQol Research Foundation, 2023). There are five response levels to the tool: no problems, slight problems, moderate problems, severe problems and extreme problems (EuroQol Research Foundation, 2019).

We also administered the EQ-Visual Analog Scale (EQ-VAS) where patients provide a global assessment of their health on a vertical visual analog scale with values between 0 (worst imaginable health) and 100 (best imaginable health) (EuroQol Research Foundation, 2019). The Filipino versions of the EQ-5D-5L and EQ-VAS have been administered in a previous cross-sectional study on a wide sample of Filipino individuals (Cheng et al., 2021). The tool has also been validated in stroke populations (Hunger et al., 2012; Golicki et al., 2015). The EQ-5D summary index for our population was derived by applying a weights to each of the levels in each dimension, with reference to the Philippine EQ-5D-5L value set published by Cheng et al. (2021).

### 2.5. Statistical analysis

Descriptive statistics were used to describe the baseline characteristics of the study population. The study participants were classified based on the HADS-P scale into: (1) no anxiety or depression, (2) with anxiety, (3) with depression, and (4) with both conditions. Distributional differences on continuous characteristics were explored using Kruskal-Wallis tests. Chi square tests were performed to assess the above four groups and their association with categorical characteristics of the study population.

Correlation coefficients and their 95% interval estimates between disability rating scales and the dimensions of the EQ-5D-5L scale were computed using the Spearman rho. Scores on the EQ-5D-5L dimensions were dichotomized into those with no problems and those with any problems (slight, moderate, severe, and extreme problems). Dichotomized dimension scores were then compared across select social and clinical demographic characteristics using chi-square and Fisher's Exact Test *p*-values.

The likelihood of having problems in the dimensions of the EQ-5D-5L scale among the participants was determined using multivariable logistic regression models. The above association was determined while accounting for the following confounders: sex, age, relationship status, employment status, educational attainment, religion, personal income, and number of caregivers. Crude associations were explored between select demographic and clinical characteristics vis-a-vis the dimension scores dichotomized into those with any or those without problems. For continuous factors, the significance testing was based on Mann–Whitney *U*-tests while chi-square and Fisher's Exact tests were used for categorical factors. Models were then created using a binary logistic regression for each dimension. All factors were factored into the model simultaneously. The adjusted odds ratio, its 95% CI, and *p*-values were determined.

## 3. Results

### 3.1. Population characteristics

A total of 1,941 stroke patients consulted at the outpatient clinics during the study period of whom 137 were between 18 and 49 years of age at the time of stroke. Of the 137, 2 patients were excluded because they exceeded the time period of 2 years after stroke, seven were excluded due to severe aphasia, six were excluded due to a low MMSE, two were excluded due to a history of depression and six were excluded due to incomplete data. A total of 114 stroke patients participated in the study with 87 patients recruited from the Neurology clinic and 27 recruited from the Rehabilitation Medicine clinic.

Health-related quality of life was measured for 114 young adult stroke patients who met the inclusion criteria. The mean age of the patients was 39.4 years. The mean duration from symptom onset to the interview was 4 months. Majority of the participants were married or had partners (71.9%) and only a third of the population attained college level education (32.5%). A large proportion of the population was unemployed and most of the population (86.8%) earned less than the monthly minimum wage in the country. Several patients (82.5%) had caregivers and spousal caregivers were most common. CVD infarct was the more common stroke subtype and these patients received antiplatelets or anticoagulants for stroke prevention. Patients who had comorbidities such as hypertension, diabetes or dyslipidemia were treated with the appropriate medications for these conditions. Almost two thirds of the population had good functional outcome (mRS 0–2) with a median Barthel Index of 95. About half of the population was found to have either anxiety, depression or both based on the HADS-P. The EQ-VAS and EQ index scores of patients without anxiety or depression were significantly higher compared to those with anxiety and/or depression (see Table 1).

**Table 1:** Baseline social and clinical demographic characteristics of the study sample.

**Characteristics**	**Overall**
Age in years	39.4 ± 7.5
Female	48 (42.1%)
**Relationship status**	
Single	24 (21.1%)
Married/with partner	82 (71.9%)
Separated/widow	8 (7.0%)
**Educational attainment**	
Elementary	7 (6.1%)
High school	55 (48.3%)
Vocational	15 (13.2%)
College level	37 (32.5%)
**Location**	
Urban	55 (48.2%)
Rural	59 (51.8%)
**Current occupation**	
Unemployed	80 (70.2%)
Employed	28 (24.6%)
**Monthly income**	
Personal monthly income	195 USD (98-293 USD)
<229 USD^*^	99 (86.8%)
≥229 USD	15 (13.2%)
**Household members**	
<5 persons	67 (58.8%)
≥5 persons	47 (41.2%)
**Social history**	
Smoking	45 (39.5%)
Alcohol abuse	57 (50%)
Illicit drug use	13 (11.4%)
**Caregivers**	
None	20 (17.5%)
Partner/spouse	35 (30.7%)
Others	24 (21.1%)
With multiple caregivers	35 (30.7%)
**Hobbies, support group membership and rehabilitation program enrollment**	
With hobbies	81 (71.1%)
With support group	5 (4.4%)
Enrolled in rehabilitation program	27 (23.7%)
**Stroke type**	
Infarct	67 (58.8%)
Intracranial hemorrhage	47 (41.2%)
**Complications**	
Post-stroke pain	19 (16.7%)
Post-stroke seizures	10 (8.8%)
**Modified Rankin scale**	
mRS 0-2	71 (62.3%)
mRS 3-4	43 (37.7%)
**MMSE**	
MMSE score	26 (24, 28)
**Barthel index**	
Barthel index score	95 (80, 100)
**Anxiety/depression based on HADS-P**	
With anxiety	30 (26.3%)
With depression	5 (4.4%)
With anxiety and depression	18 (15.8%)
**EQ-5D-5L scores**	
EQ-5D-5L index score	0.78 (0.63, 0.90)
EQ-5D-5L VAS score	80 (60, 90)

* 229 USD (12,000 PHP) is the minimum monthly wage in the country.

### 3.2. Association between the presence of anxiety and depression and the EQ-5D-5L dimensions

Table 2 shows the distribution of problem severity in the EQ-5D-5L dimensions stratified based on the presence of anxiety and depression as evaluated using the HADS-P. Among stroke survivors without anxiety or depression, a significantly higher proportion reported no problems in three dimensions of the EQ-5D-5L, namely in the dimensions of usual activities, pain or discomfort and anxiety or depression. The latter result, albeit redundant, reflects the ability of the HADS-P scale to predict anxiety and depression on the EQ-5D-5L scale.

**Table 2:** EQ-5D-5L scores among stroke survivors stratified to have anxiety and depression.

**EQ-5D-5L scales**	**No anxiety or depression (*n*, %)**	**With anxiety (*n*, %)**	**With depression (*n*, %)**	**With anxiety and depression (*n*, %)**	***p*-value**
**Mobility**					
No problems	31 (50.8%)	9 (30%)	1 (20%)	7 (39%)	0.37
Slight problems	14 (23%)	8 (26.6%)	2 (40%)	2 (11.1%)	
Moderate problems	11 (18%)	6 (20%)	2 (40%)	5 (27.8%)	
Severe problems	2 (3.3%)	2 (6.7%)	–	1 (5.6%)	
Extreme problems	3 (4.9%)	5 (16.7%)	–	3 (16.7%)	
**Self-care**					
No problems	41 (67.2%)	15 (50%)	2 (40%)	6 (33.3%)	0.13
Slight problems	10 (16.4%)	4 (13.3%)	1 (20%)	5 (27.8%)	
Moderate problems	5 (8.2%)	6 (20%)	1 (20%)	4 (22.2%)	
Severe problems	2 (3.3%)	2 (6.7%)	1 (20%)	–	
Extreme problems	3 (4.9%)	3 (10%)	–	3 (16.7%)	
**Usual activities**					
No problems	33 (54.1%)	6 (20%)	2 (40%)	3 (16.7%)	0.01^*^
Slight problems	15 (24.6%)	9 (30%)	–	4 (22.2%)	
Moderate problems	8 (13.1%)	8 (26.7%)	2 (40%)	4 (22.2%)	
Severe problems	3 (4.9%)	4 (13.3%)	1 (20%)	2 (11.1%)	
Extreme problems	2 (3.3%)	3 (10%)	–	5 (27.8%)	
**Pain or discomfort**					
No problems	25 (41%)	10 (33.33%)	2 (40%)	3 (16.67%)	0.05^*^
Slight problems	27 (44.3%)	14 (46.67%)	1 (20%)	7 (38.89%)	
Moderate problems	6 (9.8%)	5 (16.67%)	1 (20%)	5 (27.78%)	
Severe problems	1 (1.6%)	1 (3.33%)	–	3 (16.67%)	
Extreme problems	2 (3.3%)	–	1 (20%)	–	
**Anxiety or depression**					
No problems	33 (54.10%)	5 (16.67%)	1 (20%)	1 (5.56%)	<0.01^*^
Slight problems	18 (29.51%)	8 (26.67%)	3 (60%)	6 (33.33%)	
Moderate problems	8 (13.11%)	13 (43.33%)	1 (20%)	7 (38.89%)	
Severe problems	1 (1.64%)	1 (3.33%)	–	2 (11.11%)	
Extreme problems	1 (1.64%)	3 (10%)	–	2 (11.11%)	
**EQ VAS and EQ Index scores**					
EQ VAS score	90 (75, 95)^†^	75 (50, 90)^†^	50 (50, 90)^†^	60 (50, 90)^†^	0.01^*^
EQ index score	0.89 (0.75, 0.94)^†^	0.74 (0.55, 0.85)^†^	0.76 (0.68, 0.89)^†^	0.64 (0.44, 0.80)^†^	<0.01^*^

* *p* < 0.05;

† interquartile range.

The median EQ-VAS rating was significantly higher among those without depression or anxiety. The same pattern was noted for the median EQ-5D-5L index scores based on the Philippine population with significantly higher index scores noted in those without anxiety or depression. A higher EQ-5D-5L value index indicates a better HRQoL.

**Figure 1:**
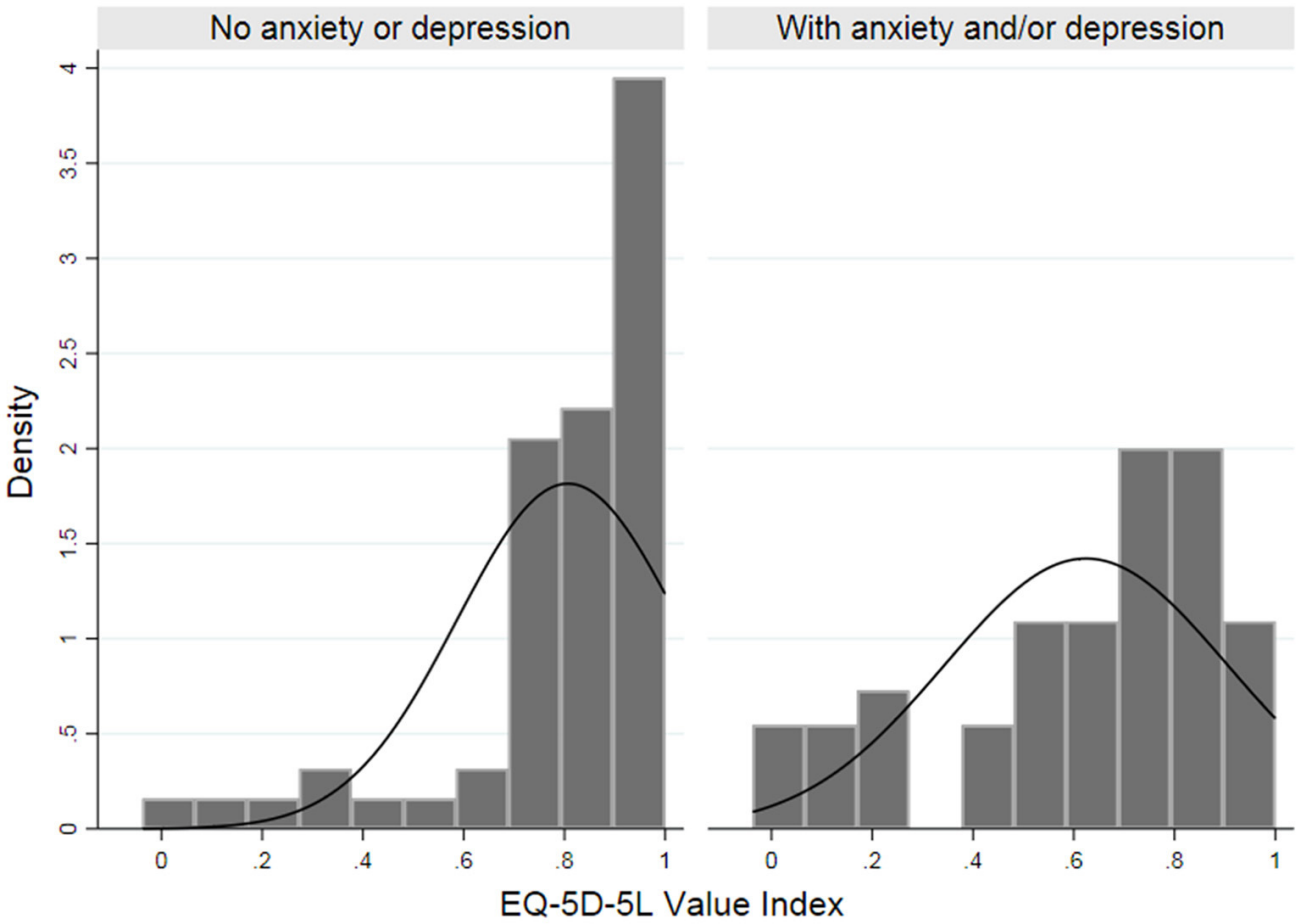
Histogram of EQ-5D-5L Index Scores between patients with and without anxiety and/or depression.

Figure 1 shows that majority of the participants without anxiety or depression scored high on the EQ-5D-5L value index with ratings greater than 0.60. This is in contrast to patients who had anxiety and/or depression showing a wider distribution of EQ-5D-5L value index ratings.

### 3.3. Correlation between EQ-5D-5L scores across other rating scales

Anxiety and depression scores on HADS-P were significantly and positively correlated with all EQ-5D-5L dimension scores. Meanwhile, mRS strongly correlated with dimensions of mobility, self-care and usual activities, while it had a moderate relationship with dimensions of pain or discomfort and anxiety or depression. Barthel Index was negatively associated with all dimensions, but the strength of its relationships was similar to that of mRS (see Supplementary Table 2).

### 3.4. Distribution of EQ-5D-5L dimensions across mRS ratings

**Figure 2:**
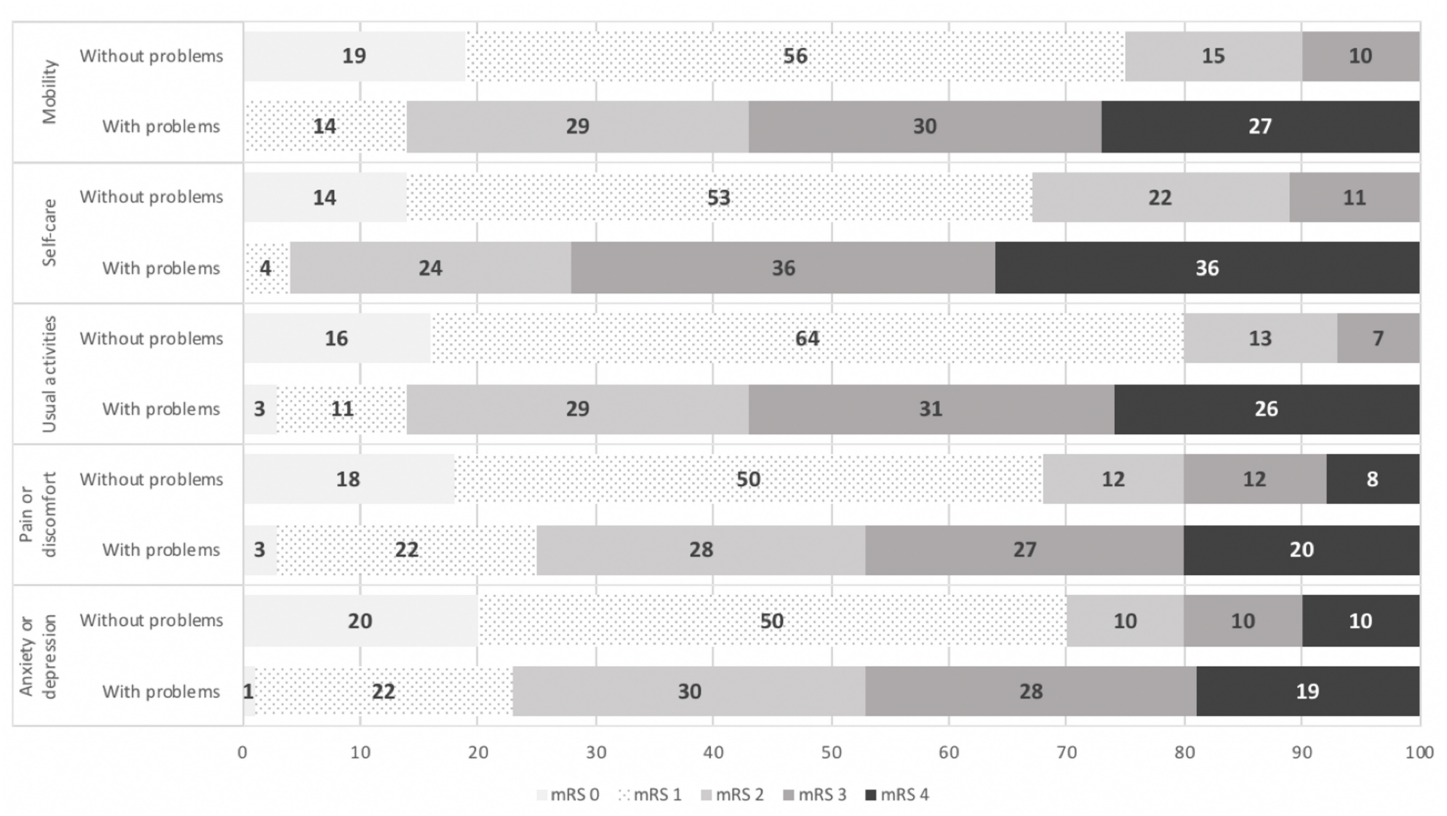
Distribution of EQ-5D-5L dimensions across mRS rating scores.

The proportion of patients who reported having no problems on all dimensions of the EQ-5D-5L was significantly higher among patients with good functional outcomes (mRS 0–2). This result is shown in Figure 2.

### 3.5. Predictors of problem severity in health-related quality of life

Multivariable regression was done to determine the correlation of the different EQ-5D-5L dimensions with various socio-economic and clinical factors. A higher mRS was associated with worse scores on all EQ-5D-5L dimensions but this did not reach statistical significance. Meanwhile, a high Barthel Index was significantly correlated with better scores on the mobility dimension (OR 0.17, 95% CI 0.03–1.02) and self-care dimension (OR 0.08, 95% CI 0.01–0.55). Intracranial hemorrhage as a stroke subtype was a predictor for poorer scores on the self-care dimension (OR 4.99, 95% CI 1.42–17.56; see Table 3).

**Table 3:** Multivariable regression analysis for significant predictors of poor HRQoL on each EQ5D5L subscale.

**Factor**	**EQ mobility**			**EQ self-care**			**EQ activities**			**EQ pain**			**EQ anxiety**		
	**OR**	**95% CI**	***p*** **-value**	**OR**	**95% CI**	***p*** **-value**	**OR**	**95% CI**	***p*** **-value**	**OR**	**95% CI**	***p*** **-value**	**OR**	**95% CI**	***p*** **-value**
Age	1.01	0.95–1.08	0.68	1.04	0.96–1.13	0.37	1.01	0.94–1.08	0.87	0.98	0.93–1.04	0.55	1.09	1.01–1.17	0.02
Anxiety	1.09	0.37–3.18	0.88	1.50	0.40–5.59	0.55	4.15	1.23–14.05	0.02^*^	1.17	0.44–3.11	0.75	11.17	3.03–41.09	<0.001
Depression	0.91	0.24–3.40	0.89	2.10	0.46–9.69	0.34	0.98	0.21–4.51	0.98	1.91	0.53–6.91	0.32	3.90	0.68–22.35	0.12
Female sex	0.71	0.27–1.86	0.49	1.89	0.58–6.12	0.29	1.26	0.43–3.68	0.67	1.21	0.50–2.92	0.67	2.38	0.84–6.74	0.10
Education			0.58			0.31			0.25			0.82			0.34
Highschool	0.72	0.12–4.22	0.72	0.30	0.03–2.89	0.30	0.11	0.01–1.23	0.07	0.59	0.09–3.80	0.58	0.51	0.05–5.60	0.58
Vocational	0.28	0.03–2.52	0.26	0.21	0.01–3.34	0.27	0.08	0.01–1.40	0.08	0.47	0.05–4.12	0.49	1.55	0.11–22.68	0.75
College	0.80	0.13–5.10	0.81	0.86	0.09–8.42	0.89	0.19	0.02–2.20	0.18	0.80	0.11–5.66	0.83	1.22	0.10–14.50	0.88
Monthly income >229 USD	0.50	0.13–1.92	0.31	0.56	0.08–4.00	0.56	0.31	0.06–1.49	0.14	0.45	0.13–1.50	0.19	0.66	0.17–2.57	0.55
mRS > 2	3.26	0.60–17.72	0.17	5.37	0.83–34.90	0.08	5.80	0.73–46.00	0.09	3.41	0.59–19.56	0.17	5.74	0.53–62.67	0.15
Barthel index ≥ 95	0.17	0.03–1.02	0.05^*^	0.08	0.01–0.55	0.01^*^	0.23	0.03–2.00	0.18	1.03	0.17–6.11	0.98	2.45	0.22–27.61	0.47
ICH stroke type	1.16	0.46–2.96	0.75	4.99	1.42–17.56	0.01^*^	1.67	0.57–4.87	0.35	1.13	0.48–2.70	0.78	2.10	0.76–5.81	0.15

* p < 0.05.

## 4. Discussion

### 4.1. Key findings

Our study showed that patients with anxiety and depression had poorer health-related quality of life reflected by lower ratings on both the EQ-VAS and EQ index scores. The presence of anxiety and depression based on the HADS-P scale was significantly correlated with poorer scores on all EQ-5D-5L dimensions. mRS and Barthel index were also found to be predictors of poor HRQoL scores. Similarly, the proportion of patients with poorer functional outcome was higher among those who reported problems on all EQ-5D-5L dimensions. Intracranial hemorrhage as a stroke subtype was identified as a risk factor for problems in the self-care dimension.

### 4.2. Impact of anxiety and depression on HRQoL

Patients with anxiety and depression in our study had lower EQ-VAS and EQ index scores compared to those without any of these conditions. Our findings are consistent with prior research reporting stroke patients with depression to have lower QOL scores (Naess et al., 2006; Kutlubaev and Hackett, 2014). Similarly, a recent study found that stroke patients with anxiety disorder had more problems on all dimensions of the EQ-5D-5L despite similar baseline neurologic impairment highlighting the impact of anxiety on a patient's quality of life (Chun et al., 2018). Stroke survivors having both anxiety and depression may have even greater impairments in social functioning as well as activities of daily living (Shimoda and Robinson, 1998; Kapoor et al., 2019). Our findings confirm the negative effect of psychiatric comorbidities on a patient's recovery and HRQoL.

### 4.3. Association of physical functioning and HRQoL

Mobility and physical functioning have been identified as independent predictors of HRQoL in stroke patients (Naess et al., 2006; Valeros, 2017; Thayabaranathan et al., 2018; Wong et al., 2021). Our study confirmed these findings showing mRS and Barthel index to have strong or moderate correlations with the various EQ-5D-5L dimensions. A higher proportion of the population with no problems on all EQ-5D-5L dimensions had good functional outcomes (mRS 0–2). Regression analysis also showed that lower Barthel Index scores, indicative of dependence on activities of daily living, were associated with worse scores on the dimensions of mobility and self-care. The correlation between mRS and Barthel index with QoL reflects the importance of mobility in activities of daily living (Joundi et al., 2021).

### 4.4. Other predictors of poor health-related quality of life

In addition to functionality and mobility, our study showed that intracranial hemorrhage was a predictor for poorer scores on the self-care dimension of the EQ-5D-5L. A recent study found that cerebral hemorrhage may be a risk factor for return to work (Chen et al., 2019). However, further research is required to establish whether stroke type is indeed an independent predictor of QoL.

Among the participants in our study, majority did not attain college level education, were unemployed and had income less than the monthly minimum wage stipulated in the country. Although these socio-economic factors were not found to be predictors of poor HRQoL in the present study, previous studies have reported female gender, low educational attainment and poor socio-economic status as part of a complex network of risk factors associated with HRQoL in stroke survivors (Naess et al., 2005; Phan et al., 2019; Wong et al., 2021). Studies with larger sample sizes are required to further evaluate these important factors and their influence on the HRQoL of young adult stroke patients.

### 4.5. HRQoL of young adult Filipino patients after stroke

Our study population's overall HRQoL as reported using the EQ VAS and EQ index scores were 80 (IQR 60–90) and 0.78 (IQR 0.63–0.90) respectively. Compared to the Philippine value set (Median 97), our population of young adult stroke patients had a lower VAS score (Cheng et al., 2021). These findings are consistent with previous studies that have reported significantly lower scores for self-rated health in stroke survivors compared to individuals who did not suffer from stroke (Xie et al., 2006).

## 5. Limitations

Our study population included participants consulting at follow-up outpatient clinics at our institution. The generalizability of our study results may thus be limited by biases in terms of socio-demographic and clinical characteristics as well as by the limited sample size. Use of the HADS-P and EQ-5D-5L tools may have also led to selection bias. These tools are self-administered, hence, patients with severe aphasia and cognitive deficits were excluded. We were also not able to evaluate certain factors such as overall family income which has been shown to be predictors of quality of life after stroke. The treatment for post stroke depression and anxiety were outside the scope of our study and we lack data regarding treatment such as anti-depressant or anti-anxiety medications.

## 6. Recommendations for future studies

Future studies should compare local populations who suffered from stroke with healthy age- and sex-matched controls. Larger multi-center studies are also required to confirm the various socio-economic and clinical predictors of HRQoL in young adult patients who suffered from stroke. These predictors should include the effect of certain treatments such as thrombolysis and endovascular thrombectomy on the HRQoL of stroke survivors. These treatments may work by reducing the neurologic deficits of stroke survivors (Joundi et al., 2021). Young adults may be especially vulnerable to poor QoL since they are left disabled during their most active and productive years (Kapoor et al., 2019; Ignacio et al., 2022). The impact of HRQoL on other outcomes such as healthcare use, social participation and response to rehabilitation should also be explored (Chun et al., 2018).

## 7. Conclusion

Our findings highlight the impact of psychiatric comorbidities on the various dimensions of HRQoL in young adults after stroke. Individuals with both anxiety and depression are particularly susceptible to poor self-rated health scores, having the lowest ratings on EQ VAS and EQ index. Poor functional outcome and dependence in activities of daily living are associated with poorer HRQoL scores on all dimensions of the EQ-5D-5L scale. Psychiatric comorbidities and assessment of mobility are important determinants of quality of life. Early identification of these conditions could prove crucial to the wellbeing and recovery of young adult Filipinos after stroke.

## Data availability statement

The original contributions presented in the study are included in the article/Supplementary material, further inquiries can be directed to the corresponding author.

## Ethics statement

The studies involving human participants were reviewed and approved by University of the Philippines Manila—Research Ethics Board through the Office of the Philippine General Hospital-Expanded Hospital Research Office (Code: 2019 270-01). The patients/participants provided their written informed consent to participate in this study.

## Author contributions

KI, JM, SS, AL, SF, SI, JP, MP-F, and JD: conceptualization, data collection, formal analysis, interpretation of data, writing—original draft, and writing—review and editing. All authors contributed to the article and approved the submitted version.

## References

[B1] Bartholomé L. Winter Y. (2020). Quality of life and resilience of patients with juvenile stroke: a systematic review. J. Stroke Cerebrovasc. Dis. 29, 10512. 10.1016/j.jstrokecerebrovasdis.2020.105129 32912563

[B2] Chen Q. Cao C. Gong L. Zhang Y. (2019). Health related quality of life in stroke patients and risk factors associated with patients for return to work. Medicine 98, e15130. 10.1097/MD.0000000000015130 31008934 PMC6494282

[B3] Cheng K. J. G. Rivera A. S. Miguel R. T. D. P. Lam H. Y. (2021). A cross-sectional study on the determinants of health-related quality of life in the Philippines using the EQ-5D-5L. Qual. Life Res. 30, 2137–47. 10.1007/s11136-021-02799-0 33677770

[B4] Chun H. Y. Y. Whiteley W. N. Dennis M. S. Mead G. E. Carson A. J. (2018). Anxiety after stroke. Stroke 49, 556–64. 10.1161/STROKEAHA.117.020078 29437982 PMC5839706

[B5] De Guzman M. L. R. E. (2013). A validation of the hospital anxiety and depression scale (HADS) in the medically-ill. Acta Med. Philipp. 47, 52–62. 10.47895/amp.v47i3.131811531728

[B6] De Ryck A. Brouns R. Fransen E. Geurden M. Van Gestel G. Wilssens I.. (2013). A prospective study on the prevalence and risk factors of poststroke depression. Cerebrovasc. Dis. Extra. 3, 1–13. 10.1159/000345557 23626594 PMC3567876

[B7] Ekker M. S. Verhoeven J. I. Vaartjes I. van Nieuwenhuizen K. M. Klijn C. J. M. de Leeuw F. E. (2019). Stroke incidence in young adults according to age, subtype, sex, and time trends. Neurology 92, e2444–54. 10.1212/WNL.0000000000007533 31019103

[B8] EuroQol Research Foundation (2019). EQ 5D-5L User Guide, 2019. Available online at: https://euroqol.org/publications/user-guides (accessed December 20, 2022).

[B9] EuroQol Research Foundation (2023). EQ-5D Registration. Available online at: https://registration.euroqol.org (accessed December 20, 2022).

[B10] Golicki D. Niewada M. Buczek J. Karlińska A. Kobayashi A. Janssen M. F.. (2015). Validity of EQ-5D-5L in stroke. Qual. Life Res. 24, 845–50. 10.1007/s11136-014-0834-1 25347978 PMC4366565

[B11] Hunger M. Sabariego C. Stollenwerk B. Cieza A. Leidl R. (2012). Validity, reliability and responsiveness of the EQ-5D in German stroke patients undergoing rehabilitation. Qual. Life Res. 21, 1205–16. 10.1007/s11136-011-0024-3 21971874

[B12] Ignacio K. H. D. Diestro J. D. B. Medrano J. M. M. Salabi S. K. U. Logronio A. J. Factor S. J. V.. (2022). Depression and anxiety after stroke in young adult filipinos. J. Stroke Cerebrovasc. Dis. 31, 106232. 10.1016/j.jstrokecerebrovasdis.2021.106232 34875539

[B13] Johnson C. O. Nguyen M. Roth G. A. Nichols E. Alam T. Abate D.. (2019). Global, regional, and national burden of stroke, 1990–2016: a systematic analysis for the Global Burden of Disease Study 2016. Lancet Neurol. 18, 439–58. 10.1016/S1474-4422(19)30034-1 30871944 PMC6494974

[B14] Joundi R. A. Rebchuk A. D. Field T. S. Smith E. E. Goyal M. Demchuk A. M.. (2021). Health-related quality of life among patients with acute ischemic stroke and large vessel occlusion in the ESCAPE trial. Stroke 52, 1636–42. 10.1161/STROKEAHA.120.033872 33691504

[B15] Kapoor A. Si K. Yu A. Y. X. Lanctot K. L. Herrmann N. Murray B. J.. (2019). Younger age and depressive symptoms predict high risk of generalized anxiety after stroke and transient ischemic attack. Stroke 50, 2359–63. 10.1161/STROKEAHA.119.025464 31405330

[B16] Kutlubaev M. A. Hackett M. L. (2014). Part II: predictors of depression after stroke and impact of depression on stroke outcome: an updated systematic review of observational studies. Int. J. Stroke 9, 1026–36. 10.1111/ijs.12356 25156411

[B17] Naess H. Nyland H. I. Thomassen L. Aarseth J. Myhr K. M. (2005). Mild depression in young adults with cerebral infarction at long-term follow-up: a population-based study. Eur. J. Neurol. 12, 194–8. 10.1111/j.1468-1331.2004.00937.x 15693808

[B18] Naess H. Waje-Andreassen U. Thomassen L. Nyland H. Myhr K. M. (2006). Health-related quality of life among young adults with ischemic stroke on long-term follow-up. Stroke 37, 1232–6. 10.1161/01.STR.0000217652.42273.02 16601213

[B19] Navarro J. C. Baroque A. C. Lokin J. K. Venketasubramanian N. (2014). The real stroke burden in the Philippines. Int. J. Stroke 9, 640–1. 10.1111/ijs.12287 24844610

[B20] Phan H. T. Blizzard C. L. Reeves M. J. Thrift A. G. Cadilhac D. A. Sturm J.. (2019). Sex differences in long-term quality of life among survivors after stroke in the INSTRUCT. Stroke 50, 2299–306. 10.1161/STROKEAHA.118.024437 31412754

[B21] Shimoda K. Robinson R. G. (1998). Effect of anxiety disorder on impairment and recovery from stroke. J. Neuropsychiatry Clin. Neurosci. 10, 34–40. 10.1176/jnp.10.1.34 9547464

[B22] Smajlovic D. (2015). Strokes in young adults: epidemiology and prevention. Vasc. Health Risk Manag. 11:157–64. 10.2147/VHRM.S53203 25750539 PMC4348138

[B23] Su S. H. Xu W. Hai J. Yu F. Wu Y. F. Liu Y. G.. (2014). Cognitive function, depression, anxiety and quality of life in Chinese patients with untreated unruptured intracranial aneurysms. J. Clin. Neurosci. 21, 1734–9. 10.1016/j.jocn.2013.12.032 24913931

[B24] Thayabaranathan T. Andrew N. E. Kilkenny M. F. Stolwyk R. Thrift A. G. Grimley R.. (2018). Factors influencing self-reported anxiety or depression following stroke or TIA using linked registry and hospital data. Qual. Life Res. 27, 3145–55. 10.1007/s11136-018-1960-y 30078162

[B25] Valeros M. (2017). Quality of life among post stroke patients at the Zamboanga City Medical Center. J. Neurol. Sci. 381, 1118. 10.1016/j.jns.2017.08.3154

[B26] von Elm E. Altman D. G. Egger M. Pocock S. J. Gøtzsche P. C. Vandenbroucke J. P. (2007). Strengthening the reporting of observational studies in epidemiology (STROBE) statement: guidelines for reporting observational studies. BMJ. 335, 806–8. 10.1136/bmj.39335.541782.AD 17947786 PMC2034723

[B27] Wong H. J. Lua P. L. Harith S. Ibrahim K. A. (2021). Health-related quality of life profiles and their dimension-specific associated factors among Malaysian stroke survivors: a cross sectional study. Health Qual. Life Outcomes 19, 210. 10.1186/s12955-021-01847-0 34461920 PMC8406972

[B28] Xie J. Wu E. Q. Zheng Z. J. Croft J. B. Greenlund K. J. Mensah G. A.. (2006). Impact of stroke on health-related quality of life in the noninstitutionalized population in the United States. Stroke 37, 2567–72. 10.1161/01.STR.0000240506.34616.10 16946158

