# Supplementary material for: Impact of post stroke depression and anxiety on health-related quality of life in young Filipino adults
Source: Front Stroke. 2023 Mar 16;2:1149406. doi: 10.3389/fstro.2023.1149406 (PMC12802660; doi:10.3389/fstro.2023.1149406)
Supplement: Supplementary file 1 [file Table_1.docx]

Supplementary Material

Impact of poststroke depression and anxiety on health-related quality of life in young Filipino adults

Katrina Hannah D. Ignacio MD, Jose Miguel M. Medrano MD, Sitti Khadija U. Salabi MD, Alvin J. Logronio, MD, Sedric John V. Factor MD , Sharon D. Ignacio MD, Jose Leonard R. Pascual V MD, Carissa Pineda-Franks, MD^1^, Jose Danilo B. Diestro MD^7^

*** Correspondence:** Katrina Hannah D. Ignacio: kdignacio@up.edu.ph

# Supplementary Tables

Supplementary Table 1. Number and percentage of participants responding on each level of the EQ-5D-5L subscales

| **Mobility** | Number (n) | Percentage |
| --- | --- | --- |
| No problems | 7 | 42% |
| Slight problems | 2 | 23% |
| Moderate problems | 5 | 21% |
| Severe problems | 1 | 4% |
| Extreme problems | 3 | 10% |
| **Self-care** |  |  |
| No problems | 6 | 56% |
| Slight problems | 5 | 18% |
| Moderate problems | 4 | 14% |
| Severe problems | 0 | 4% |
| Extreme problems | 3 | 8% |
| **Usual activities** |  |  |
| No problems | 3 | 38% |
| Slight problems | 4 | 25% |
| Moderate problems | 4 | 19% |
| Severe problems | 2 | 9% |
| Extreme problems | 5 | 9% |
| **Pain/discomfort** |  |  |
| No problems | 3 | 35% |
| Slight problems | 7 | 43% |
| Moderate problems | 5 | 15% |
| Severe problems | 3 | 4% |
| Extreme problems | 0 | 3% |
| **Anxiety/depression** |  |  |
| No problems | 1 | 35% |
| Slight problems | 6 | 31% |
| Moderate problems | 7 | 25% |
| Severe problems | 2 | 4% |
| Extreme problems | 2 | 5% |

Supplementary Table 2. Spearman’s Correlation between EQ-5D-5L scores across other Rating Scales

| **Spearman's rho** | **EQ Mobility** | **EQ Self-care** | **EQ Activities** | **EQ Pain** | **EQ Anxiety** |
| --- | --- | --- | --- | --- | --- |
| HADS-A | .196* | .279** | .372** | .231* | .573** |
| HADS-D | .205* | .246** | .323** | .247** | .483** |
| HADS Total | .226* | .295** | .388** | .266** | .581** |
| mRS | .715** | .746** | .684** | .467** | .461** |
| Barthel Index Score | -.713** | -.840** | -.696** | -.393** | -.447** |
| EQ VAS | -0.171 | -.207* | -.294** | -.227* | -.368** |

**correlation is significant at the 0.01 level

*correlation is significant at the 0.05 level
